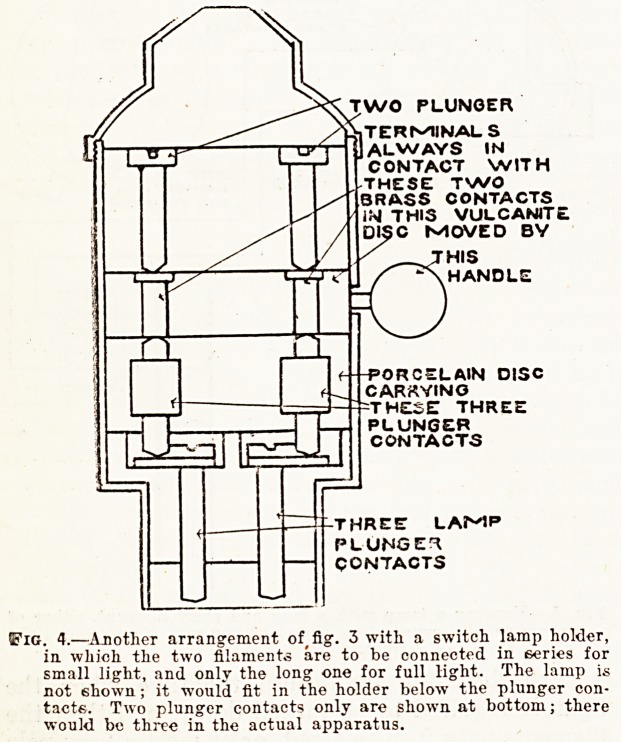# The Lighting of Wards.—I

**Published:** 1912-05-11

**Authors:** 


### PRACTICAL POINTS.

(Criticism and Suggestions Invited.)

### The Lighting of Wards.—I.

### HOW TO ARRANGE FOR TURNING ELECTRIC LIGHTS DOWN AT NIGHT.

THE incandescent electric light has become practically universal in all up-to-date hospitals, and there can be no doubt that it is very much better in every way than gas. It presents one difficulty, however, which gas does not. Gas can be turned down as much as you please; with electric light

FIG. 1.—Arrangement for turning down the light of an incandescent lamp, or group of lamps, by means of a graduated resistance inserted in the circuit.

it is not so easy. The ordinary incandescent electric lamp cannot be turned down at all, without a special switch to which a resistance is attached, an arrangement that increases the expense very considerably, complicates the fixing, and increases the chances of breakdown. Further, the current

FIG. 2.—Showing the connections for switching three lamps in parallel, so that they will give their full light and in series when they give a red or dull yellow light. When the switch bar is in position shown no current is passing at all. When the switch bar is turned to the right, to the first position, the current passes through the lamps in series, when it is over in the right-hand position the current divides between the lamps. There is no electrical connection to the blank stop shown.

which passes through the resistance when the light is turned down is wasted. Fig. 1 shows this arrangement.

There have been several devices placed upon the market designed to overcome the difficulty. In one method three lamps are arranged in a group, and a three-way switch is attached to the fitting, or a separate switch is fixed in some convenient position near. With the switch in one position the current is completely cut off; with the switch in a second position, the three lamps are connected in parallel circuit so that they give their full light; and in a third position the three lamps are connected in series, so that the resistance of the three being interposed across the service, in place of that of one lamp, the current passing through all the lamps is very much reduced, and the light with it. With this arrangement the three lamps are made to

FIG. 3.—Showing a lamp with a long and short filament, either of which can be switched on, the short one giving a smaller light and taking less current.

furnish their full light until the time when the light is required to be turned down, and then the filaments only furnish a red, or at most a very pale yellow, glow. This arrangement is also wasteful, because the current is flowing through the whole of the lamps during the whole of the lighting period, and for practical purposes is uselessly heating two of the lamps, while the light given is not at all a useful one. Fig. 2 shows the connections for this arrangement.

In another and perhaps more convenient method that has been introduced for the same purpose there are two distinct filaments in one lamp globe, and the lamp is controlled by a three-way switch, on somewhat similar lines to that of the three-lamp arrangement. When the switch is turned to one contact, the current is cut off; when it is turned to a second, the current passes through the filament while a good light is wanted; when the switch is turned to the third contact, the current passes through the two filaments so that it furnishes a certain amount of light, just sufficient for night work, when it is connected in series with the day filament. This also is wasteful, because the current is heating the longer filament uselessly during the whole of the hours when the short one is giving light.

In a later form of lamp for night work the two filaments are quite independent of each other, the smaller one being made of very much higher resistance, and therefore to give a very much smaller amount of light. It is a much shorter filament, but also very much thinner, and can therefore only be made for comparatively low voltages. The switching arrangement is practically the same. The current may be cut right off, it may be directed through the large filament for light during the evening, and through the small filament for light at night. This is a more economical arrangement than either of the others, but it has the drawback that it is difficult

Fig. 4.—Another arrangement of fig. 3 with a switch lamp holder, in which the two filaments are to be connected in series for small light, and only the long one for full light. The lamp is not shown; it would fit in the holder below the plunger contacts. Two plunger contacts only are shown at bottom; there would be three in the actual apparatus.

to construct a satisfactory three-way switch within the limits of the lamp-holder, or, if the switch is fixed separately, to arrange connections sufficiently strong for the purpose. Figs. 3 and 4 show the arrangement. Fig. 4 is a switch lamp-holder arranged to turn on either filament.

### A New Departure.

A new departure has recently been made, which promises very favourably, both for convenience and for economy. By its means lamps of varying candle-power can be employed, and can be turned in and out at will, so that a lamp of low candle-power can be used at night, while one or more lamps of higher candle-power are used during the evening. It also has the advantage that metallic filament lamps of low pressure can be used. The metallic filament lamp, it will be remembered, takes only about one-third of the current, with any given pressure, that is required by the carbon filament lamp, to furnish the same light. The metallic filament lamp, however, has suffered from one drawback; it has been difficult to make low candle-power filaments for the service pressures which have ruled for some years past in all large towns. Some years ago the engineers who controlled the electricity generating stations exerted pressure upon the manufacturers of carbon filament incandescent lamps, and obliged them to produce lamps that would work at from 200 to 260 volts. The production of these lamps of higher pressures enabled the number of lamps upon any distribution service, in any town, to be very largely increased, four times and upwards, without increasing the size of the cables. The metallic filament lamp, as is well known, consists of a very fine metallic wire, made from the rare metals tantalum, osmium, tungsten, and others. It is exceedingly difficult to draw very fine wires of these metals, and as the higher the voltage with which the lamp is to work, and the lower the candle-power, the finer the filament has to be, the difficulty will be easily apparent. Metallic filament lamps for 10 volts, 25 volts, 50 volts, and even 100 volts, for low candle-power, are made with comparative ease, and their filaments are strong. The lower the voltage for a given candle-power the stronger necessarily is the filament. Under the pressure of the supply engineers, the manufacturers of metallic filament lamps have produced lamps of comparatively low candle-power to work with the higher pressures, 200 to 250 volts, but they are more expensive, they do not last so long, they take more current, and they are generally more delicate. They have not been made so far for smaller lamps than 16 candle-power.

In the new method that has been introduced the electrical condenser is employed. This consists of a number of sheets of metal, usually tin-foil or some similar substance, separated by sheets of insulating material. In the common form, the separating medium is paraffined paper, the conductors being tin-foil. In modern condensers, and particularly in those that are employed in the new departure for electric lamps to be described, the construction of the condenser has been greatly improved. Paraffined paper is a somewhat unreliable substance; it is apt to be sparked through, and then the condenser is useless. This difficulty has been overcome in the apparatus to be described, which is only applicable for use with alternating currents; but as in almost every town where electricity is now used for lighting, alternating currents are employed, owing to the ease and economy with which large areas can be covered, it is probable that there are very few hospitals where the apparatus will not be of service. Even where continuous currents are employed, they could easily be converted to alternating currents for supplying the lamps mentioned.

*(To be continued.)*

## Figures and Tables

**Fig. 1. f1:**
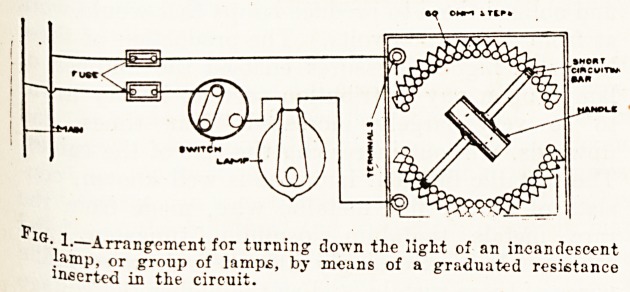


**Fig. 2. f2:**
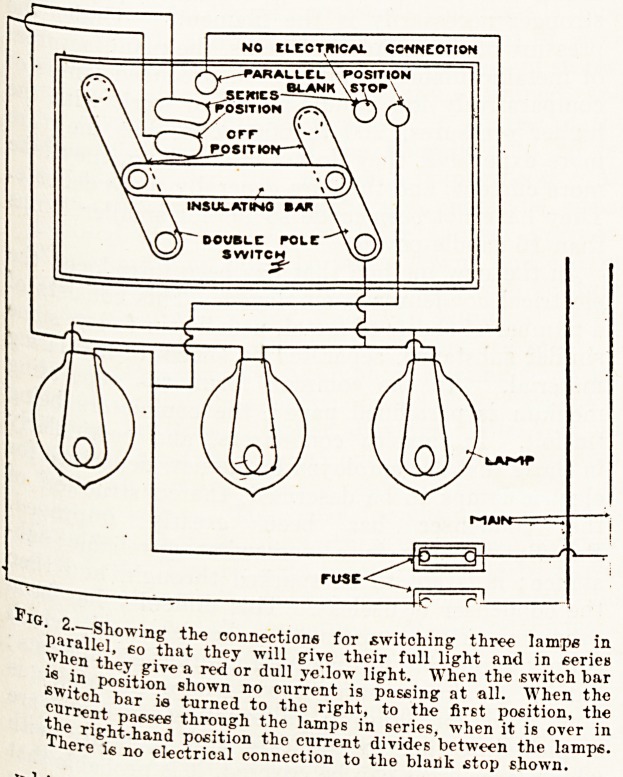


**Fig. 3. f3:**
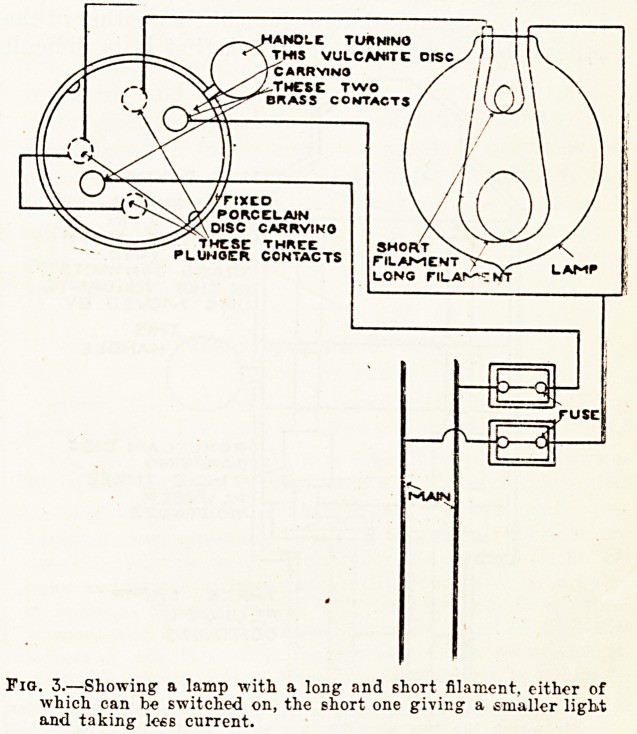


**Fig. 4. f4:**